# Gastric atrophy: use of OLGA staging system in practice 

**Published:** 2016

**Authors:** Mahsa Molaei, Ara Ehtiati, Reza Mashayekhi, Mitra Rafizadeh, Homayoun Zojaji, Dariush Mirsattari, Roya Kishani Farahani

**Affiliations:** 1 *Pathology Department, Gastroenterology and Liver Diseases Research Center, Research Institute for Gastroenterology and Liver Diseases, Shahid Beheshti University of Medical Sciences, Tehran, Iran*; 2 *Department of Pathology, Shahid Beheshti University of Medical Sciences, Tehran, Iran*; 3 *Basic and Molecular Epidemiology of Gastroenterology Disorders Research Center, Research Institute for Gastroenterology and Liver Diseases, Shahid Beheshti University of Medical Sciences, Tehran, Iran*

**Keywords:** Chronic gastritis, Atrophy, Intestinal metaplasia, Staging

## Abstract

**Aim:**

This study used the OLGA system to characterize the histology pattern of gastritis in dyspeptic outpatients with a mean age of 45 years from regions with different gastric cancer risks.

**Background:**

Several classification systems have been purposed for understanding the status of the gastric mucosa. Currently, the Sydney system is the most widely employed. Nevertheless, the applicability of the Sydney system in therapeutic and prognostic areas is a matter of debate. Given this shortcoming an international group of gastroenterologists and pathologists developed a new system named Operative Link on Gastritis Assessment (OLGA).

**Patients and methods:**

In this cross-sectional comparative study the OLGA system was used to characterize the histology pattern of gastritis in 685 dyspeptic patients referring to the department of gastroenterology of a training hospital.

**Results:**

No significant correlation was found between active inflammation and total OLGA score (P > 0.05). Also, no statistically significant correlation was found between activity and intestinal metaplasia, dysplasia, atrophy, and cancer (P > 0.05). Even though, there is a positive correlation between mild chronic inflammation and total OLGA score, no correlation has been identified between chronicity and dysplasia or cancer (P > 0.05). Nearly, In all cases with no dysplasia *OLGA score* was *zero but all patients with gastric cancer *OLGA score was more than two.

**Conclusion:**

Generally, the activity is not a useful factor in predicting prognosis and its loss of relation with total OLGA score does not make OLGA score any less predictable.

## Introduction

*H.pylori* associated or epidemic gastric cancer lies in the downstream of a succession of genetic and phenotypic events, which is triggered by *H.pylori* infection (1). Evidences from previous researches illustrated the link of gastric cancer and *H.pylori* associated chronic gastritis and the subsequent atrophy of the gastric mucosa. It has been suggested that the risk of cancerogenesis in gastric mucosa can be predicted based on the severity of succeeding outcomes of *H.pylori* infection (2-4). In this era, several classification systems have been introduced for a better understanding of the gastric mucosa status (4-6). The Updated Sydney System is currently the most widely employed system that conveys information on topographic, morphologic, and microbiological aspects of the diseased gastric mucosa. The Updated Sydney System also provides more consistency in comparison to its predecessors (5-7).However, the applicability of the Updated Sydney System either in therapeutic or prognostic areas is a matter of debate (8). 

Although Sydney System is widely cited, its recommendations regarding the format of biopsy reporting have been taken for granted to a large extent, and more emphasis has been put on its four-point grading system of histological lesions (8). Moreover, using the Updated Sydney System, the cancer risk can hardly be elicited even by well-trained specialists based upon “multi- focal atrophic gastritis”, “corpus predominant gastritis”, or “antrum-predominant *H. pylori* chronic active gastritis” (6, 9, 10).

Given these shortcomings, an international group of gastroenterologists and pathologists (Operative Link on Gastritis Assessment [OLGA]) developed a new system for histological reporting of gastritis (2, 7). The OLGA system incorporated the semi-quantitative scoring systems established by the Sydney System and the subsequent Atrophy International Club Guidelines and expresses the extent/location of atrophic/metaplastic lesions in terms of gastritis staging (11). Therefore, the reported gastritis stage encompasses the atrophy extent and topographic information that are derived from histological scoring and anatomic lesion mapping. This study used the OLGA system to characterize the histology pattern of gastritis in dyspeptic outpatients with a mean age of 45 years from regions with different gastric cancer risks. 

## Patients and Methods

In this cross-sectional comparative study, 685 dyspeptic patients referred to the gastroenterology department of a training hospital between 2012 and 2013 were evaluated. Clinical data (history and physical examination) were retrieved by a trained general practitioner, who also took informed consent from participants. To maximize the coherency of endoscopic performance (gross evaluation and tissue sampling) one adroit gastroenterologist in upper GI endoscopy was assigned to perform the procedure. The study protocol was approved by the ethics committee of the Research Institute of Gastroenterology and Liver Diseases, Shahid-Beheshti University.

Patients with previous history of upper GI surgery were excluded from the study. Patients in whom the endoscopy was successful, and had yielded satisfactory amounts of the gastric tissue sample were included in the study. The implemented biopsy sampling protocol was that of Sydney Staging System (12, 13). Therefore, at least five tissue specimens were obtained from the gastric mucosa of each patient, including two from antral mucosa, two from corpus, and one sample from incisura angularis. Samples were fixed (in 5-10% formalin), stored in separate vials and labeled according to their site before transferring to the department of pathology. Any abnormal lesion in stomach or duodenum was noted and reported during endoscopy as well, and convenient samples were taken accordingly.

Formalin-fixed tissue samples were embedded in paraffin. Three sections (4 micron thick) were obtained from each block, and stained in advance with Hematoxylin and Eosin (H&E), as well as Periodic Acid-Schiff (PAS), and Giemsa. To preserve the anonymity of patients, samples and blindness of examining pathologists, slides were coded, labeled, and set randomly in this phase. Two pathologists, who were blinded and deprived from any clinical data regarding participants and the location of the samples, examined the slides mutually and issuance of a report entailed a mutual consensus on each item by the examining pathologists. 

**Figure 1:**
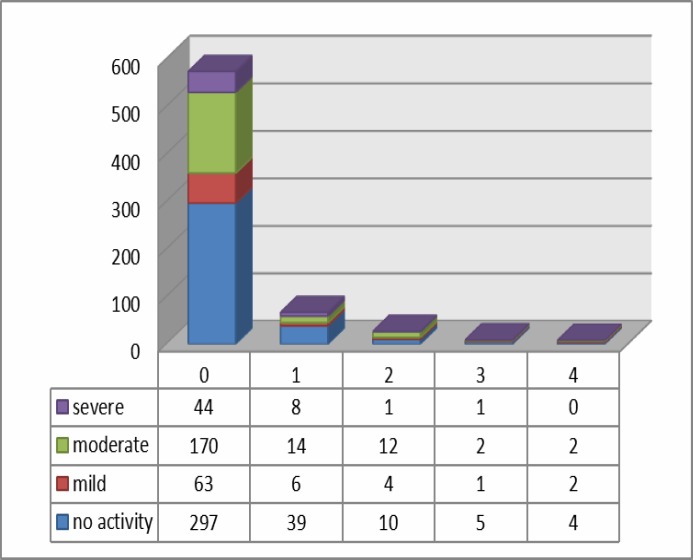
Antrum-active inflammation*Total OLGA score

The atrophy status of each slide was determined as atrophic or non-atrophic. Atrophy was defined as loss of glandular structures with or without epithelial metaplasia (12, 14). The extent of atrophy was reported in advance using a visual analogue scale according to the Sydney Staging System (12, 13); 0=absent, 1= mild, 2=moderate, 3=severe. Considering the severity and site of atrophy (corpus, antrum/incisura angularis), the gastritis stage was calculated according to the OLGA international proposal (Figure 1 and Tables 1-3) (8, 15). Moreover, the subjects were considered *H. pylori* positive (HP+) if the bacteria could be detected histologically in any of Giemsa-stained slides (rods on the epithelial surface). Thus, negative *H. pylori* status (HP-) represented the lack of histological detection of *H. pylori* in Giemsa-stained samples.

Data analysis was performed among 685 patients by SPSS (version 13.0) software [Statistical Procedures for Social Sciences; Chicago, Illinois, USA] using Kruskal-Wallis and Chi-Square tests. P<0.05 was considered to indicate a statistically significant difference. 

## Results

The mean (± standard deviation) age of patients were 45.26 ± 17.56 years, ranging from 12 to 87 years. Also the mean OLGA score was 0.26 ± 0.70 ranging from 0 to 4. Among females, 50.5% and among male subjects 49.5% had OLGA score zero. Positive correlation was found between the antrum versus body chronicity and total OLGA score. Patients with more severity had higher total OLGA scores (P values 0.005 and 0.002, respectively). No correlation was found between the antrum activity and total OLGA score (P > 0.5); but positive correlation was seen between the body activity and total OLGA score (P=0.001).

There was a positive correlation between the antrum versus body dysplasia and total OLGA score (P=0.0001). Also a positive correlation was found between the antrum versus body cancer and total OLGA score (P=0.001) (table 2, 3). No correlation was found between endoscopic duodenal ulcer and total OLGA score (P > 0.5). No correlation was found between antrum versus body activity and intestinal metaplasia, dysplasia or cancer (P > 0.5) (table 4, 5). 

**Table 1:** The association of body - active inflammation and total OLGA score

		Total OLGA score (gastritis stage)				
Body - active inflammation		0	1	2	3	4
	no activity	336	39	11	3	5
	mild	93	5	3	2	0
	moderate	124	15	12	3	3
	severe	21	8	1	1	0

**Table 2:** Association of antrum-dysplasia and total OLGA score

		Total OLGA score (gastritis stage)				
Antrum - Dysplasia		0	1	2	3	4
	absent	573	66	21	8	8
	present	1	1	6	1	0

**Table 3:** Association of body dysplasia and total OLGA score

		Total OLGA score (gastritis stage)				
Body - Dysplasia		0	1	2	3	4
	absent	574	66	24	9	6
	present	0	1	3	0	2

## Discussion

According to the information from patients, in cases with antrum and body chronic inflammation a positive correlation is present with a total OLGA score and most relation has been found in mild inflammation. Most cases with mild inflammation or no inflammation had the total OLGA score of zero. However, in cases with moderate to severe chronic inflammation total OLGA score was above zero without any other sort of relation.

There is no positive correlation between antrum or body activity and total OLGA score. By going through the information that has been mentioned in the analysis part, we understand that there is also no relation between these factors and cancer, dysplasia or metaplasia. In conclusion, it can be said that activity is not a useful prognostic factor and its loss of relation with total OLGA score does not make OLGA score any less predictable.

There were not any association between the scores 4 to 1 in *H.pylori* infection; therefore the *H.pylori* infection status is always mentioned with OLGA score (positive or negative). The most relationship between intestinal metaplasia and total OLGA score were seen at the zero score. Due to this reason, all patients who did not have intestinal metaplasia had a zero score and all patients who were suffered from intestinal metaplasia scored above zero. 

In antrum biopsies the OLGA score is raising the ratio of positive intestinal metaplasia but turns more towards negative, so that at score of 4 the ratio will reach 8. Meaning, all 8 patients with the score of 4 have intestinal metaplasia.

**Table 4:** Association of Antrum - Cancer and total OLGA score

		Total OLGA score (gastritis stage)				
		0	1	2	3	4
Antrum - Cancer	absent	574	67	25	9	8
	present	0	0	2	0	0

**Table 5:** Association of Body - Cancer and total OLGA score

		Total OLGA score (gastritis stage)				
		0	1	2	3	4
Body - Cancer	absent	574	67	25	8	7
	present	0	0	2	1	1

In body biopsies with the increase of score the ratio of cases with positive intestinal metaplasia will shift towards more negative, but this increase is not categorized appropriately. In case of the antrum, the most relation between body atrophy and total OLGA score can be seen in the zero score. However, the increase of atrophy is not leveled with the increase of scores at higher scores. Most Patients with dysplasia have not OLGA score, but zero score has been reported. In all patients with cancer the OLGA score was higher than 2.

Our results showed that patients with mild chronic gastritis have lower total OLGA scores (score 0 versus 1). The total score of patients with severe and moderate chronic gastritis is more than 1. Antrum activity and body activity have no correlation with a total OLGA score, as well as dysplasia or cancer. Therefore, reporting activity in the Sydney system cannot help us to predict prognosis. All patients with dysplasia in antrum or body have total OLGA scores more than zero.

All patients showing cancer in antrum or body have total OLGA scores over 2. There is no correlation between antrum or body activity and dysplsia/cancer. There is no correlation between antrum or body chronocity and dysplsia/cancer. Therefore, these findings that are basic items in the Sydney system cannot be used to estimate patient’s prognosis, but total OLGA score have a good correlation with dysplasia and cancer.

In 2003, the Chinese Society of Digestive Endoscopy established endoscopic criteria for chronic gastritis in Dalian meeting. The scar lesions were characterized by the following attributes: mucosal atrophy, granular mucosa, flattened folds, gray intestinal-type epithelium and blood vessel permeability (16).
